# *Burkholderia pseudomallei* in Soil, US Virgin Islands, 2019

**DOI:** 10.3201/eid2611.191577

**Published:** 2020-11

**Authors:** Nathan E. Stone, Carina M. Hall, A. Springer Browne, Jason W. Sahl, Shelby M. Hutton, Ella Santana-Propper, Kimberly R. Celona, Irene Guendel, Cosme J. Harrison, Jay E. Gee, Mindy G. Elrod, Joseph D. Busch, Alex R. Hoffmaster, Esther M. Ellis, David M. Wagner

**Affiliations:** Northern Arizona University, Flagstaff, Arizona, USA (N.E. Stone, C.M. Hall, J.W. Sahl, S.M. Hutton, E. Santana-Propper, K.R. Celona, J.D. Busch, D.M. Wagner);; US Virgin Islands Department of Health, Charlotte Amalie, US Virgin Islands, USA (A.S. Browne, I. Guendel, C.J. Harrison, E.M. Ellis);; Centers for Disease Control and Prevention, Atlanta, Georgia, USA (A.S. Browne, J.E. Gee, M.G. Elrod, A.R. Hoffmaster)

**Keywords:** Burkholderia pseudomallei, melioidosis, environmental detection, soil, Virgin Islands, US Virgin Islands, hurricanes, bacteria, United States

## Abstract

The distribution of *Burkholderia pseudomallei* in the Caribbean is poorly understood. We isolated *B. pseudomallei* from US Virgin Islands soil. The soil isolate was genetically similar to other isolates from the Caribbean, suggesting that *B. pseudomallei* might have been introduced to the islands multiple times through severe weather events.

*Burkholderia pseudomallei* is a gram-negative soil-dwelling bacterium and the causative agent of melioidosis (*1*). *B. pseudomallei* is endemic to tropical regions around the world (*1*), but its environmental distribution in the Caribbean remains poorly understood. Although it is rare but ecologically established in Puerto Rico (*2*,*3*), it has not been isolated from the environment in the neighboring US Virgin Islands (USVI). After the 2017 Caribbean hurricane season, melioidosis developed in 3 persons in the USVI (*4*), 2 in St. Thomas and 1 in St. John. We aimed to determine whether, as this cluster suggests, *B. pseudomallei* might be endemic to the USVI.

We collected 480 soil and 100 freshwater samples from 29 sites (24 terrestrial and 5 freshwater) on the 3 main USVI islands (i.e., St. Thomas, St. John, and St. Croix) during January 20–April 17, 2019. We selected study sites to maximize geographic distribution across the islands and epidemiologic connection to melioidosis cases in humans (Appendix Figure 1). These efforts followed consensus guidelines for environmental sampling of *B. pseudomallei* (*5*) and methods previously reported (*2*) with 4 modifications: we collected 20 samples per site; we collected soil samples in 2 linear transects of 10 samples each; we collected 150 mL water per sample; and we used half of each sample for our analysis (the other half was archived). Although we strove for a sampling depth of 30 cm in soil, this was impossible at some sites because of rocks and debris (Appendix Table 1). We placed environmental samples in Ashdown’s liquid media for *Burkholderia* spp. enrichment (*2*). After enrichment, we extracted DNA using QiaAmp kits (QIAGEN, https://www.qiagen.com) and screened it using a *B. pseudomallei*–specific TaqMan assay (ThermoFisher Scientific, https://www.thermofisher.com) (*6*,*7*). We cultured samples to isolate pure *B. pseudomallei* and generate whole-genome sequences (WGSs). We conducted a phylogenetic analysis as previously described (*2*) and conducted genetic typing on these WGSs, WGSs from the 3 patients with melioidosis from USVI in 2017, and 43 additional *B. pseudomallei* WGSs available in GenBank from the Caribbean, the Americas, and Africa (Appendix Table 2).

We isolated *B. pseudomallei* from only 1 (»4%) of 24 soil sites, a prevalence resembling that of nearby Puerto Rico (*2*), where another study isolated *B. pseudomallei* from 2 soil samples collected at only 1 (2%) of 50 sampled sites. We obtained the *B. pseudomallei*–positive sample from site 122 (Appendix Figure 1), which was adjacent to a paved roadway 76 meters above sea level on eastern St. John. We collected the soil sample, which was composed of dry gravelly loam and had a pH of 6.9, from a depth of 30 cm (*8*) (Appendix; Appendix Table 1, Figure 2).

Our phylogenetic analysis assigned the 4 isolates (3 from patients, 1 from the environment) from the USVI to a monophyletic clade with all other *B. pseudomallei* isolates from the Caribbean (except 1 from Aruba) (Figure). However, none of the 4 isolates from the USVI were close genomic matches. These isolates differed by 6,355–10,115 single-nucleotide polymorphisms (SNPs) in the core genome, exhibiting more genomic diversity than *B. pseudomallei* isolates within Puerto Rico and Martinique (Figure). The 2019 soil and 2017 human isolates from St. John were not closely related (differing by 10,115 core genome SNPs), suggesting multiple introductions of *B. pseudomallei* to this island. The closest genomic match to the St. John soil isolate (differing by 170 core genome SNPs) was a 2007 isolate from Road Town, Tortola, British Virgin Islands (*9*). Although the dispersal mechanism of *B. pseudomallei* to this region is unknown, a dispersal event between these 2 locations (»11 km) might have been caused by aerosolization of *B. pseudomallei* during an extreme weather event, such as a hurricane (*10*). This mechanism of long-distance dispersal might also explain why the 2017 isolate from St. John is more closely related to isolates from Martinique than to the other isolates from USVI; this patient from the USVI was infected shortly after hurricane Maria (*4*). We placed the 2 isolates, despite differing by 6,355 core genome SNPs, from patients on St. Thomas in a single subclade; this pattern might suggest long-term endemicity on this island. However, these scenarios are based on an analysis of a relatively small number of *B. pseudomallei* WGSs from the Caribbean.

**Figure:**
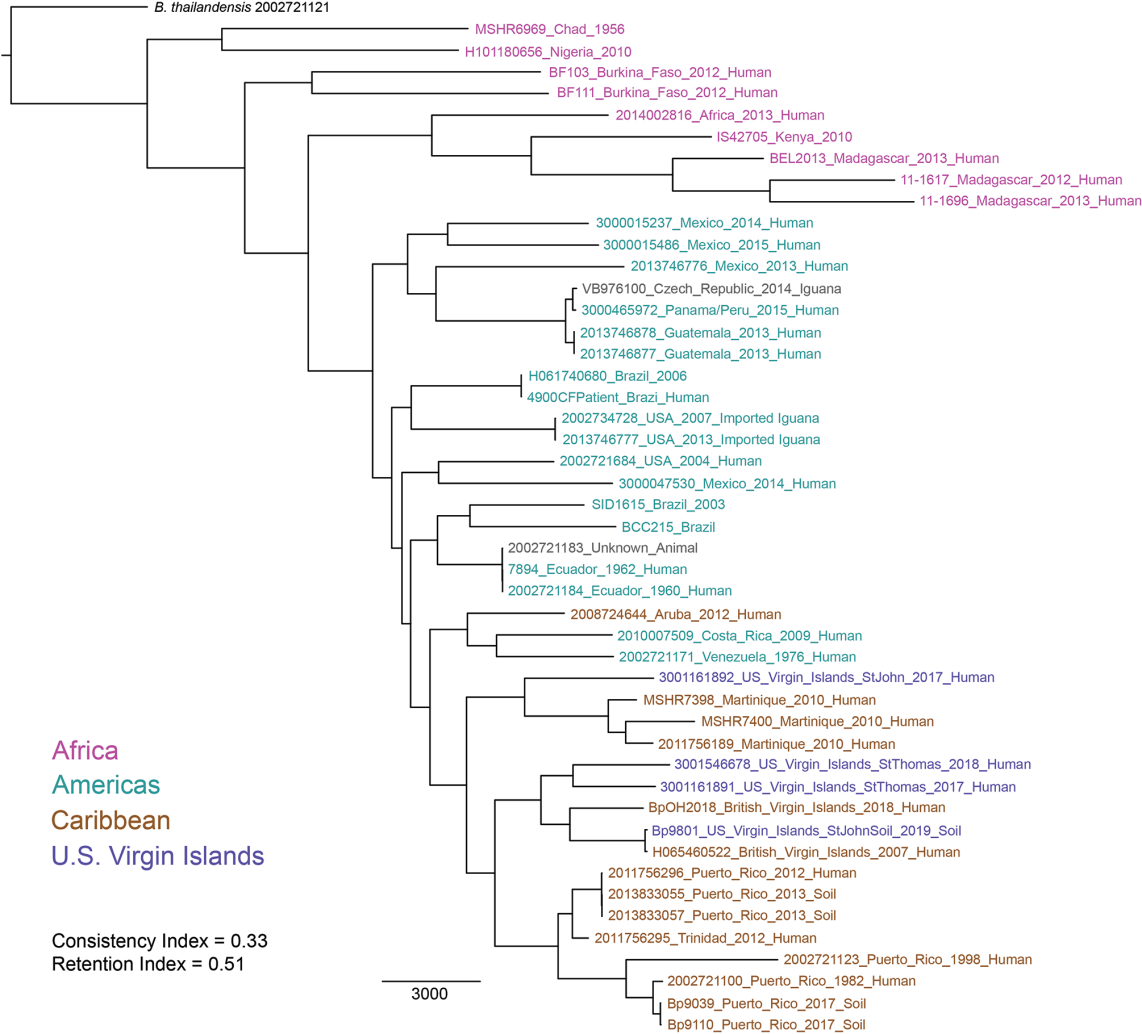
Maximum-likelihood phylogeny of *Burkholderia pseudomallei* isolates from patients and the environment in the US Virgin Islands and reference isolates available in GenBank from other countries in the Americas, Africa, and the Caribbean.

Our findings demonstrate that *B. pseudomallei* is rare in the environment in the USVI. The 2017 cases of melioidosis and the soil isolate from St. John indicate this bacterium might be ecologically established in the USVI. Additional environmental sampling will determine the environmental distribution of *B. pseudomallei* in the USVI, aiding the development of public health strategies to mitigate the risk for melioidosis.

Appendix Additional information about soil samples collected in the US Virgin Islands, 2019.
