# Supplementary material for: Burkholderia pseudomallei in Soil, US Virgin Islands, 2019
Source: Emerg Infect Dis. 2020 Nov;26(11):2773–5. doi: 10.3201/eid2611.191577 (PMC7588534; doi:10.3201/eid2611.191577)
Supplement: Appendix — Additional information about soil samples collected in the US Virgin Islands, 2019. [file 19-1577-Techapp-s1.pdf]

Article DOI: https://doi.org/10.3201/eid2611.191577

Burkholderia pseudomallei in Soil, US Virgin Islands, 2019

Appendix

Description of *Burkholderia pseudomallei*–Positive Soil Sample

The soil type and soil profile for this sample were very gravelly loam (Appendix Figure 2) and southgate-rock outcrop complex, respectively (Appendix Table 1). Southgate has a typical geologic profile of 0–5 inches: gravelly loam; 5–10 inches: very gravelly loam; 10–17 inches: weathered bedrock; and 17–60 inches: unweathered bedrock. The southgate landform is hillsides, mountain slopes, or ridges, and it is well drained with no flooding. Rock outcrop has a typical profile of 0–60 inches: unweathered bedrock, and a landform of ridges. These soil characteristics and landform information were obtained from the USDA National Resource Conservation Service's Web Soil Survey (*1*).

References:

1. US Department of Agriculture. Web Soil Survey [cited 2019 Sep 16]. https://websoilsurvey.sc.egov.usda.gov/

2. Liguori AP, Warrington SD, Ginther JL, Pearson T, Bowers J, Glass MB, et al. Diversity of 16S-23S rDNA internal transcribed spacer (ITS) reveals phylogenetic relationships in *Burkholderia pseudomallei* and its near-neighbors. PLoS One. 2011;6:e29323. PubMed https://doi.org/10.1371/journal.pone.0029323

3. Tuanyok A, Auerbach RK, Brettin TS, Bruce DC, Munk AC, Detter JC, et al. A horizontal gene transfer event defines two distinct groups within *Burkholderia pseudomallei* that have dissimilar geographic distributions. J Bacteriol. 2007;189:9044–9. PubMed https://doi.org/10.1128/JB.01264-07

4. Tuanyok A, Stone JK, Mayo M, Kaestli M, Gruendike J, Georgia S, et al. The genetic and molecular basis of O-antigenic diversity in *Burkholderia pseudomallei* lipopolysaccharide. PLoS Negl Trop Dis. 2012;6:e1453. PubMed https://doi.org/10.1371/journal.pntd.0001453

5. Sitthidet C, Stevens JM, Chantratita N, Currie BJ, Peacock SJ, Korbsrisate S, et al. Prevalence and sequence diversity of a factor required for actin-based motility in natural populations of *Burkholderia* species. J Clin Microbiol. 2008;46:2418–22. PubMed https://doi.org/10.1128/JCM.00368-08

6. Sahl JW, Caporaso JG, Rasko DA, Keim P. The large-scale blast score ratio (LS-BSR) pipeline: a method to rapidly compare genetic content between bacterial genomes. PeerJ. 2014;2:e332. PubMed https://doi.org/10.7717/peerj.332

7. Rasko DA, Myers GS, Ravel J. Visualization of comparative genomic analyses by BLAST score ratio. BMC Bioinformatics. 2005;6:2. PubMed https://doi.org/10.1186/1471-2105-6-2

Appendix Table 1. Sampling sites for *Burkholderia pseudomallei*, US Virgin Islands, 2019*

Site ID	Sample type	No. samples collected	Collection date	Island	Elevation, m	pH at site			Soil sampling depth, cm	USDA soil profile	Observed soil profile	Soil moisture
						Average	Max	Min				
106	Soil	20	2019 Jan 20	St. Thomas	143.60	5.82	6.30	5.30	30	Very gravelly clay loam	Sandy loam, clay	Dry
107	Soil	20	2019 Jan 21	St. Thomas	21.04	7.70	9.80	6.30	30	Very gravelly loam	Sandy clay loam	Dry
108	Soil	20	2019 Jan 21	St. Thomas	10.06	8.47	9.10	6.70	20–30	Silt loam	Sandy clay loam	Moist/wet/soaked
109	Soil	20	2019 Jan 22	St. Thomas	14.63	8.78	9.70	7.30	30	Very gravelly loam	Sandy clay	Dry
110	Soil	20	2019 Jan 22	St. Thomas	10.06	8.72	9.10	8.20	15–30	NL	Sandy loam, loam	Dry/moderately moist/moist
111	Soil	20	2019 Jan 22	St. Thomas	10.06	8.66	9.40	8.20	15–20	Very gravelly clay loam	Sand, sandy loam	Moist
112	Soil	20	2019 Jan 23	St. Thomas	12.80	8.48	9.10	7.20	30	Clay loam	Sandy loam	Moderately moist
113	Freshwater	20	2019 Jan 21	St. Thomas	26.52	8.72	8.80	8.50	NA	NA	NA	NA
114	Freshwater	20	2019 Jan 21	St. Thomas	10.37	7.80	7.80	7.70	NA	NA	NA	NA
115	Soil	20	2019 Jan 23	St. Thomas	12.50	9.19	9.50	8.60	30	Gravelly fine sandy loam	Sandy loam	Moist
116	Soil	20	2019 Jan 24	St. Thomas	30.79	7.39	8.50	6.30	20–30	Very gravelly loam	Loam, sandy clay loam	Dry/moderately moist
117	Soil	20	2019 Jan 28	St. John	10.37	8.22	9.20	7.10	30	Very gravelly loam	Clay, sandy clay loam, sandy loam	Moderately moist
118	Soil	20	2019 Jan 28	St. John	7.93	9.42	9.90	8.60	30	NL	Loam, sandy clay loam	Dry/moderately moist/moist
119	Soil	20	2019 Jan 28	St. John	12.20	7.23	9.80	6.60	30	Gravelly clay loam	Loam	Moderately moist
120	Soil	20	2019 Jan 29	St. John	37.20	8.49	9.50	5.60	30	Very gravelly clay loam	Loam	Dry/moderately moist
121	Freshwater	20	2019 Jan 28	St. John	11.28	7.93	8.20	7.90	NA	NA	NA	NA
122	Soil	20	2019 Jan 29	St. John	75.91	7.8	8.7	6.7	20–30	Very gravelly loam	Sandy loam	Dry/moderately moist
123	Soil	20	2019 Jan 30	St. John	9.76	8.09	8.80	7.30	30	Clay loam	Clay loam	Moist
124	Soil	20	2019 Jan 30	St. John	9.76	7.76	9.20	6.40	30	Very gravelly loam	Clay	Moist

Site ID	Sample type	No. samples collected	Collection date	Island	Elevation, m	pH at site			Soil sampling depth, cm	USDA soil profile	Observed soil profile	Soil moisture
						Average	Max	Min				
149	Freshwater	20	2019 Apr 16	St. Croix	31.10	8.04	8.20	7.80	NA	NA	NA	NA
150	Soil	20	2019 Apr 15	St. Croix	16.16	8.61	9.20	7.90	30	Clay	Loam	Dry
151	Freshwater	20	2019 Apr 16	St. Croix	73.17	8.30	8.40	8.10	NA	NA	NA	NA
152	Soil	20	2019 Apr 15	St. Croix	25.30	9.03	9.40	8.50	30	Clay	Loam	Dry
153	Soil	20	2019 Apr 15	St. Croix	43.90	9.29	9.70	7.70	30	Clay loam	Clay loam	Dry
154	Soil	20	2019 Apr 16	St. Croix	11.89	9.15	9.80	8.10	30	Clay	Clay loam	Dry
155	Soil	20	2019 Apr 16	St. Croix	28.96	8.73	9.50	7.80	30	Very gravelly clay	Gravels	Dry
156	Soil	20	2019 Apr 17	St. Croix	18.60	8.99	9.70	7.00	30	Very gravelly loam	Gravels	Dry
157	Soil	20	2019 Apr 17	St. Croix	30.49	8.87	9.50	7.80	30	Very gravelly clay	Clay	Dry
158	Soil	20	2019 Apr 17	St. Croix	22.26	7.47	8.90	6.60	30	Very gravelly clay	Gravels	Dry

*NA, not applicable; NL, not listed; min, minimum; max, maximum; USDA, US Department of Agricutlure (*1*).

Appendix Table 2. *Burkholderia pseudomallei* genomes from GenBank*

Strain	Country	Year	Isolate source	GenBank accession no.	Multilocus sequence type	ITS type (*2*)	Presence of YLF and BTFC gene clusters (*3*)	LPS type† (*4*)	Observed variant of *bimA*‡ (*5*)	Biosample no.
MSHR6969	Chad	1956	NA	ERR298347	ST82	C	YLF	A	*bimA-Bm*	SAMEA1920075
H101180656	Nigeria	2010	NA	ERR298772	ST707	C	YLF	A	*bimA-Bm*	SAMEA1920046
BF103	Burkina Faso	2012	Human	SRR3145394	ST1121	G	YLF	A	*bimA-Bm*	SAMN04376904
BF111	Burkina Faso	2012	Human	SRR3145395	ST1122	C	YLF	A	*bimA-Bm*	SAMN04376905
2014002816	Africa	2013	Human	GCA_002113945	ST1053	C	YLF	A	*bimA-Bm*	SAMN06007575
IS42705	Kenya	2010	NA	ERR351984	ST1540	E	YLF	A	*bimA-Bm*	SAMEA2163737
BEL2013	Madagascar	2013	Human	SRR3145396	ST1043	E	YLF	A	*bimA-Bm*	SAMN04376903
11–1617	Madagascar	2012	Human	SRR3145392	ST1054	C	YLF	Negative	*bimA-Bm*	SAMN04376901
11–1696	Madagascar	2012	Human	SRR3145393	ST1053	C	YLF	Negative	*bimA-Bm*	SAMN04376902
3000015237	Mexico	2014	Human	GCA_002111045	ST951	G	YLF	A	neither	SAMN06007576
3000015486	Mexico	2015	Human	GCA_002111385	ST92	G	YLF	A	*bimA-Bm*	SAMN06007577
2013746776	Mexico	2013	Human	GCA_002111185	ST297	G	YLF	A	neither	SAMN06007568
VB976100	Czech Republic	2014	Iguana	GCA_001885195	ST436	G	YLF	A	*bimA-Bm*	SAMN05930279
3000465972	Panama/Peru	2015	Human	GCF_002111305	ST436	G	YLF	A	*bimA-Bm*	SAMN06007579
2013746878	Guatamala	2013	Human	GCA_002111005	ST1038	G	YLF	A	neither	SAMN06007572
2013746877	Guatamala	2013	Human	GCA_002111245	ST1038	G	YLF	A	neither	SAMN06007571
H061740680	Brazil	2006	NA	ERR298754	ST92	G	YLF	A	*bimA-Bm*	SAMEA1920028
4900CFPatient	Brazil	NA	Human	GCA_000648355	ST92	G	YLF	A	*bimA-Bm*	SAMN02044373
2002734728	USA	2007	Iguana	GCA_002111105	ST518	G	YLF	A	*bimA-Bm*	SAMN06007559
2013746777	USA	2007	Iguana	GCA_002111205	ST518	G	YLF	A	*bimA-Bm*	SAMN06007569

Strain	Country	Year	Isolate source	GenBank accession no.	Multilocus sequence type	ITS type (*2*)	Presence of YLF and BTFC gene clusters (*3*)	LPS type† (*4*)	Observed variant of *bimA*‡ (*5*)	Biosample no.
2002721684	USA	2004	Human	GCA_002110925	ST297	G	YLF	A	*bimA-Bm*	SAMN06007558
3000047530	Mexico	2014	Human	GCA_002111285	ST92	G	YLF	A	*bimA-Bm*	SAMN06007578
SID1615	Brazil	2003	NA	ERR298753	ST92	G	YLF	A	*bimA-Bm*	SAMEA1920029
BCC215	Brazil	NA	NA	GCA_000170595	ST1355	G	YLF	A	*bimA-Bm*	SAMN02470671
2002721183	Unknown	NA	Animal	GCA_001976195	ST11	G	YLF	A	neither	SAMN04208558
7894	Ecuador	1962	Human	GCA_000959265	ST11	G	YLF	A	neither	SAMN03075630
2002721184	Ecuador	1960	Human	GCA_002111085	ST11	G	YLF	A	neither	SAMN06007557
2008724644	Aruba	2012	Human	GCA_002115385	ST698	G	YLF	A	*bimA-Bm*	SAMN06007560
2010007509	Costa Rica	2009	Human	GCA_002111145	ST518	G	YLF	A	*bimA-Bm*	SAMN06007564
2002721171	Venezuela	1976	Human	GCA_002111345	ST12	G	YLF	A	*bimA-Bm*	SAMN06007556
3001161892	St. John, US Virgin Islands	2017	Human	GCA_003584055	ST92	G	YLF	A	*bimA-Bm*	SAMN09941299
MSHR7398	Martinique	2010	Human	ERR298357	ST92	G	YLF	A	*bimA-Bm*	SAMEA1920085
MSHR7400	Martinique	2010	Human	ERR298359	ST92	G	YLF	A	*bimA-Bm*	SAMEA1920088
2011756189	Martinique	2010	Human	GCA_002111165	ST92	G	YLF	A	neither	SAMN06007574
3001546678	St. Thomas, US Virgin Islands	2018	Human	GCA_007995115	ST1492	G	YLF	A	*bimA-Bm*	SAMN12500044
3001161891	St. Thomas, US Virgin Islands	2017	Human	GCA_003584065	ST951	G	YLF	A	*bimA-Bm*	SAMN09941298
BpOH2018	British Virgin Islands	2018	Human	SRS5472236	ST92	G	YLF	A	*bimA-Bm*	SAMN12905775
Bp9801	St. John, US Virgin Islands	2019	Soil	SRR10148518	ST1492	G	YLF	A	*bimA-Bm*	SAMN12784135
H065460522	British Virgin Islands	2007	Human	ERR298778	ST1492	G	YLF	A	*bimA-Bm*	SAMEA1920053
2011756296	Puerto Rico	2012	Human	GCA_002110985	ST297	G	YLF	A	*bimA-Bm*	SAMN06007567
2013833055	Puerto Rico	2013	Soil	GCA_002111025	ST297	G	YLF	A	*bimA-Bm*	SAMN06007573
2013833057	Puerto Rico	2013	Soil	GCA_002111265	ST297	G	YLF	A	*bimA-Bm*	SAMN06007574
2011756295	Trinidad	2012	Human	GCA_002110965	ST297	G	YLF	A	*bimA-Bm*	SAMN06007566
2002721123	Puerto Rico	1998	Human	GCA_002111325	ST92	G	YLF	A	*bimA-Bm*	SAMN06007555
2002721100	Puerto Rico	1982	Human	GCA_002111065	ST95	G	YLF	A	*bimA-Bm*	SAMN06007554
Bp9039	Puerto Rico	2017	Soil	SRR7457394	ST297	G	YLF	A	*bimA-Bm*	SAMN09073285
Bp9110	Puerto Rico	2017	Soil	SRR7457423	ST297	G	YLF	A	*bimA-Bm*	SAMN09073352

**bimA*, *Burkholderia* intracellular motility A gene; BTFC, *B. thailandensis*–like flagellum and chemotaxis; ITS, 16S–23S internal transcribed spacer; LPS, lipopolysaccharide; LS-BSR, large-scale Blast Score Ratio; NA, not available; YLF, *Yersinia*-like fimbrial. Genome assemblies were screened for genotypes by using the LS-BSR method (*6*) with a 0.95 BSR (*7*) threshold.
†GenBank gene references: LPS A (BPSL2676), LPS B (BBK_2267), and LPS B2 (BTI_920).
‡GenBank gene references: *bimA-Bp* (BURPS668_A2118), *bimA-Bm* (BPSS1492).

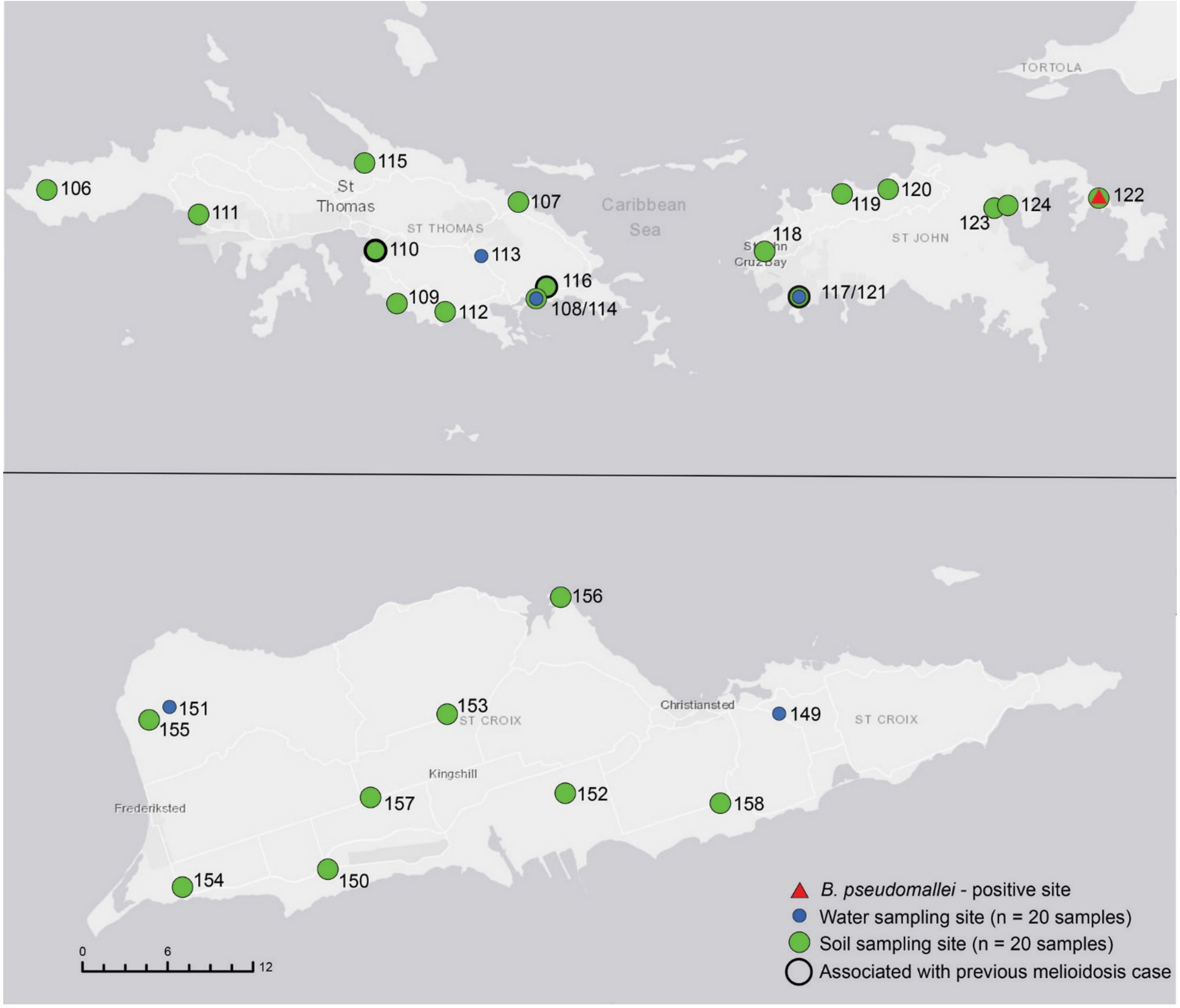

Appendix Figure 1. Sampling sites for *Burkholderia pseudomallei*, US Virgin Islands, 2019. Numbers indicated site IDs. Map created with ArcGIS software by Esri.

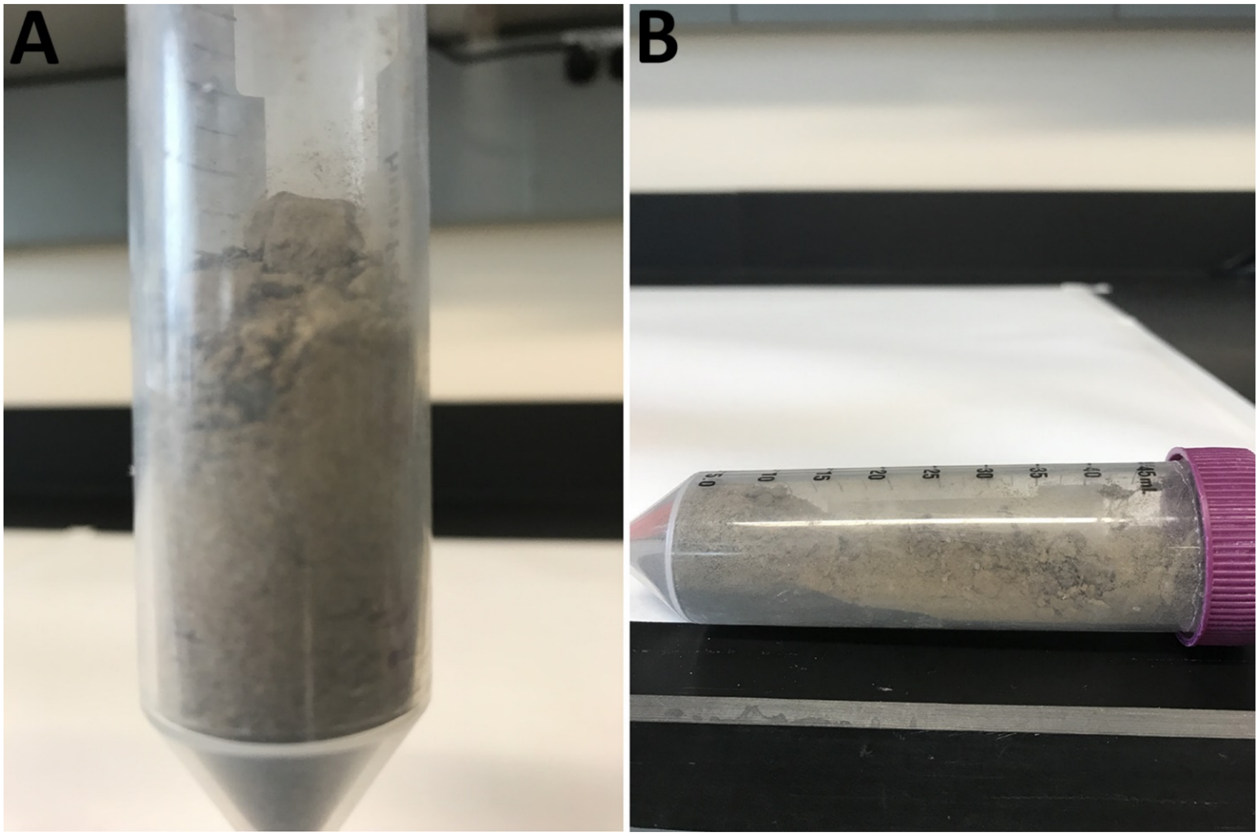

Appendix Figure 2. Photographs of *Burkholderia pseudomallei*–positive soil sample, US Virgin Islands, 2019.
